# Supplementary material for: Pyruvate dehydrogenase operates as an intramolecular nitroxyl generator during macrophage metabolic reprogramming
Source: Nat Commun. 2023 Aug 22;14:5114. doi: 10.1038/s41467-023-40738-4 (PMC10444860; doi:10.1038/s41467-023-40738-4)
Supplement: Supplementary file 1 — Supplementary Information [file 41467_2023_40738_MOESM1_ESM.pdf]

Supplementary Information

Pyruvate Dehydrogenase Operates as an Intramolecular Nitroxyl Generator During Macrophage Metabolic Reprogramming

Erika M. Palmieri[1], Ronald Holewinski[2], Christopher L. McGinity[1], Ciro L. Pierri[3], Nunziata Maio[4], Jonathan M. Weiss[1], Vincenzo Tragni[3], Katrina M. Miranda[5], Tracey A. Rouault[4], Thorkell Andresson[2], David A. Wink[1], and Daniel W. McVicar[1]*

1. Cancer Innovation Laboratory, NCI-Frederick MD 21702, USA
2. Protein Characterization Laboratory, Frederick National Laboratory for Cancer Research, Leidos Biomedical Research, Inc., Frederick, MD 21702, USA.
3. Laboratory of Biochemistry, Molecular and Structural Biology, Department of Pharmacy-Pharmaceutical Sciences, University of Bari, Via E. Orabona, 4, 70125 Bari, Italy.
4. Molecular Medicine Branch, Eunice Kennedy Shriver National Institute of Child Health and Human Development, 9000 Rockville Pike, Bethesda, MD 20892, USA
5. Department of Chemistry and Biochemistry, University of Arizona, Tucson, AZ 85721, USA

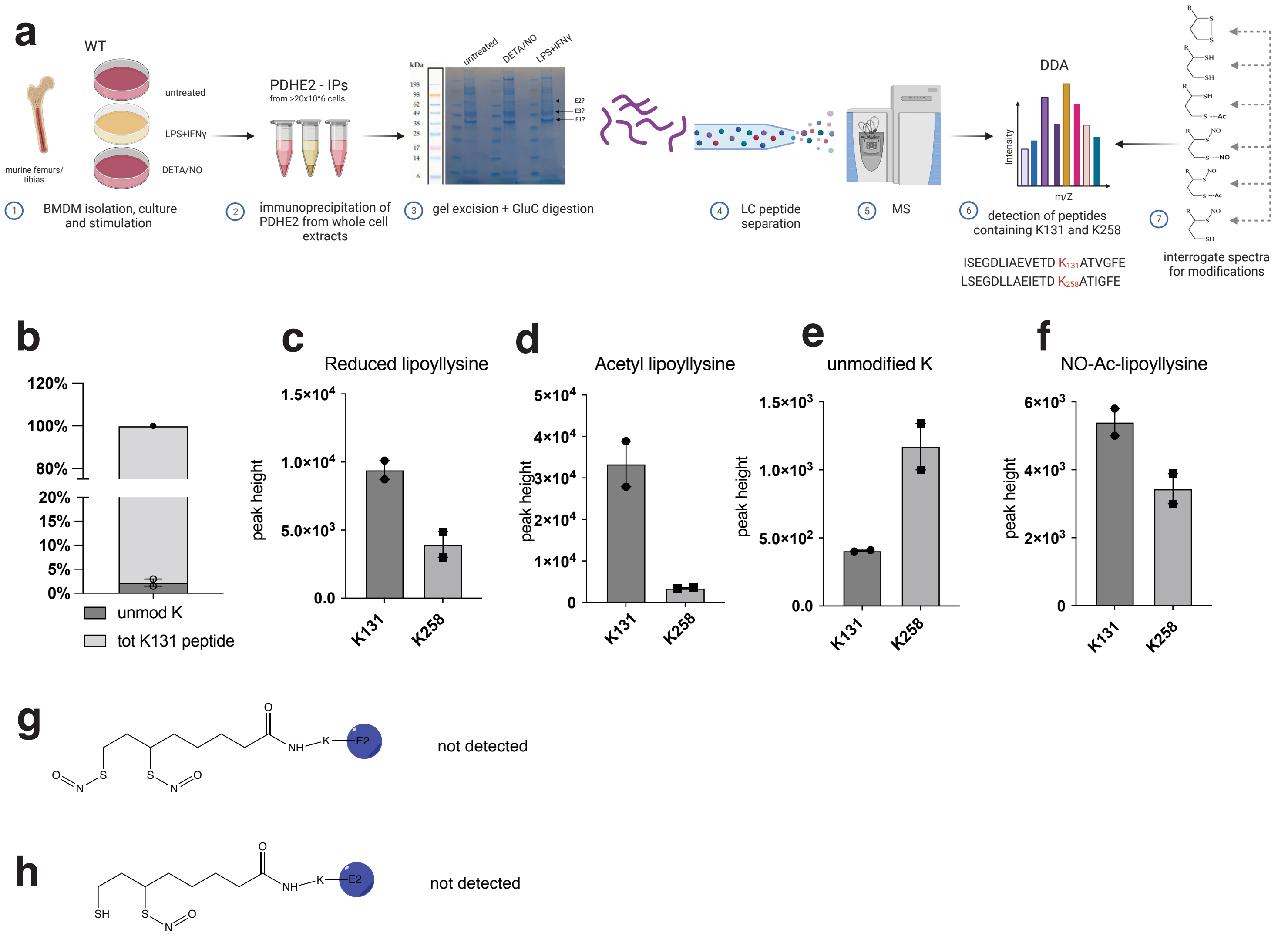

Supplementary Figure 1: a Experimental design for proteomics of E2 in BMDMs-derived lysates with a focus on lipoate containing peptides. **b** Stacked bar graph representing quantification of portion of the lypoyl-modified PDH-E2 peptide containing K^{131}. **c-f** Differences in levels of lypoyl modifications in E2 K^{131} and K^{258}, both predicted to bind lipoate cofactor. Data show abundances of E2 peptides in mitochondrial lysates from WT BMDMs from male mice (n = 2 technical repeats). **g-h** Undetected interrogated products and possible structures of E2 K^{131}-lipoylated peptide in mitochondrial lysates from WT BMDMs after 16h stimulation with LPS+ IFNγ or DETA/NO (500μM). All error bars display mean ± SEM. Data shown are representative of 2 or more independent experiments. Source data are provided as a Source Data file.

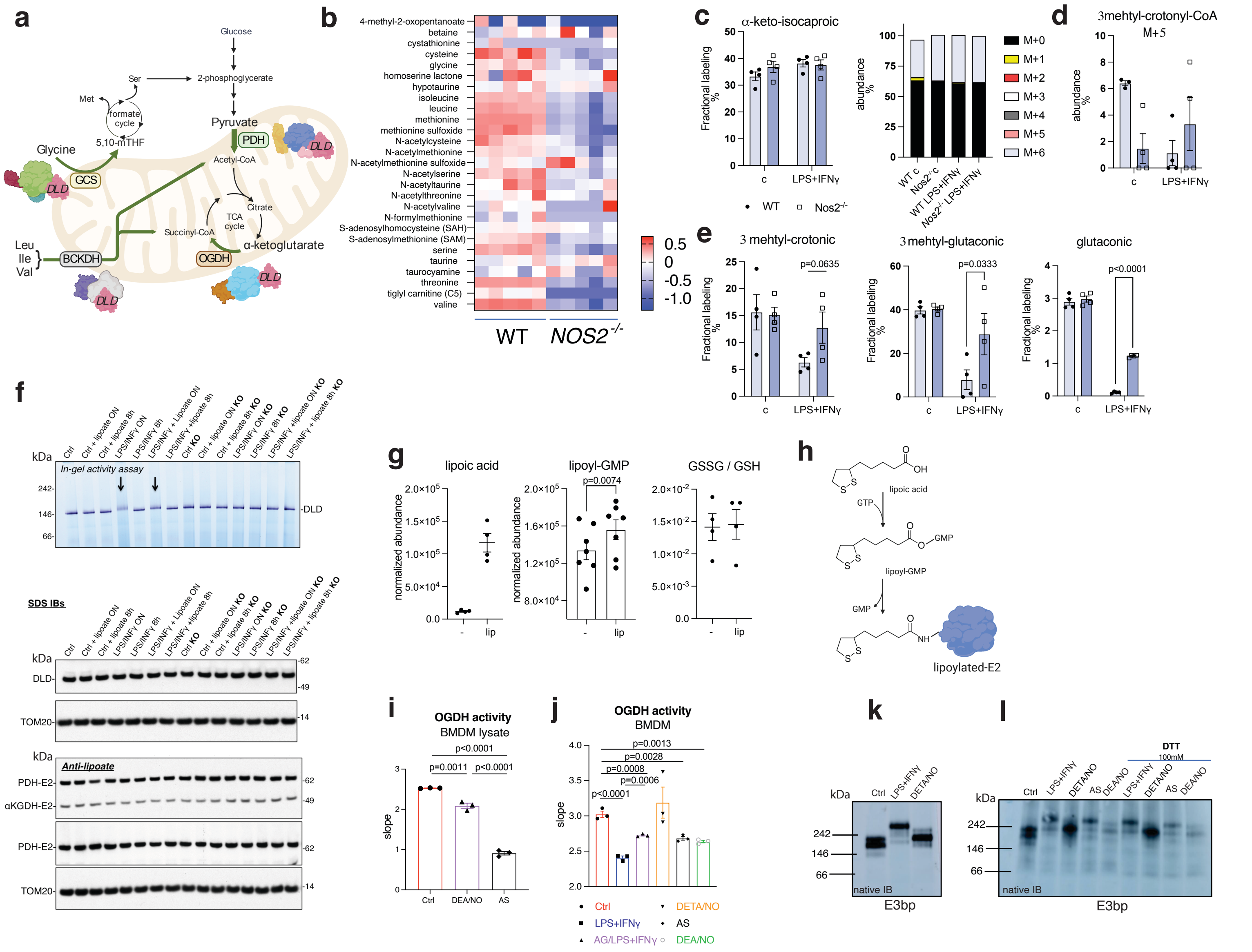

Supplementary Figure 2: a Schematic illustration of enzymes sharing E3 (DLD). GCS Glycine Cleavage System, BCKDH Branched Chain α-ketoacid Dehydrogenase, OGDH oxoglutarate dehydrogenase. **b** Heat-maps of log10 ratio from areas of metabolites associated with OGDH, BCKD, GCS from BMDMs from WT and *Nos2*$^{-/-}$ male and female mice (mixed genders in all groups) activated with LPS+ IFNγ for 16h (n = 5 mice). **c** BMDMs were activated as in **b** and cultured with [U-13C]-Leucine for additional 4h. Bars show 13C-leucine-derived carbon incorporation into M+ 0-6 isotopologues of α-ketoisocaproic acid, derived from leucine transamination (n = 4 mice). **d-e** % abundance of M+5 isotopologue and fractional enrichment of metabolites downstream of BCKDH (n = 4 mice) in BMDMs from **c,** (two-way ANOVA with Sidak's post-tests). **f** DLD in-gel activity and native IBs on mitochondrial fractions from BMDMs from WT and *Nos2*$^{-/-}$ untreated or stimulated LPS+ IFNγ for 8h or 16h (n = 9 total mice). Correspondent SDS IBs are shown. Treatments with lipoate were performed 2h before harvest. **g** WT BMDMs were treated as in **f**: metabolites were quantified at ESI-LC/MS (n > 3 mice) (paired t test, two-tailed). **h** Illustration of salvage pathway of lipoic acid in mammals. **i** BMDMs mitochondrial lysates were treated with AS or DEA/NO and OGDH activity was assayed (n = 3 mice). **j** WT BMDMs were stimulated for 16h with LPS+ IFNγ or pretreated with AG for 1 h or treated with AS or DEA/NO for 4h, and OGDH activity was assayed (n = 3 mice) (one way ANOVA with Tukey's (**i**) and Dunnet's (**j**) multiple comparisons test). **k-l** Native E3bp-IB on mitochondrial fractions from BMDMs after 16h stimulation with LPS+ IFNγ or DETA/NO (500μM), or with AS or DEA/NO for 4h. Lysates pre-native PAGE were incubated with 100mM DTT (n = 8 total mice). All error bars display mean ± SEM. Data shown are representative of 2 or more independent experiments except (**g** middle panel) which are cumulative data from 2 independent experiments. p values > 0.05 are reported as "ns" unless otherwise specified. Source Data are provided as a Source Data file.

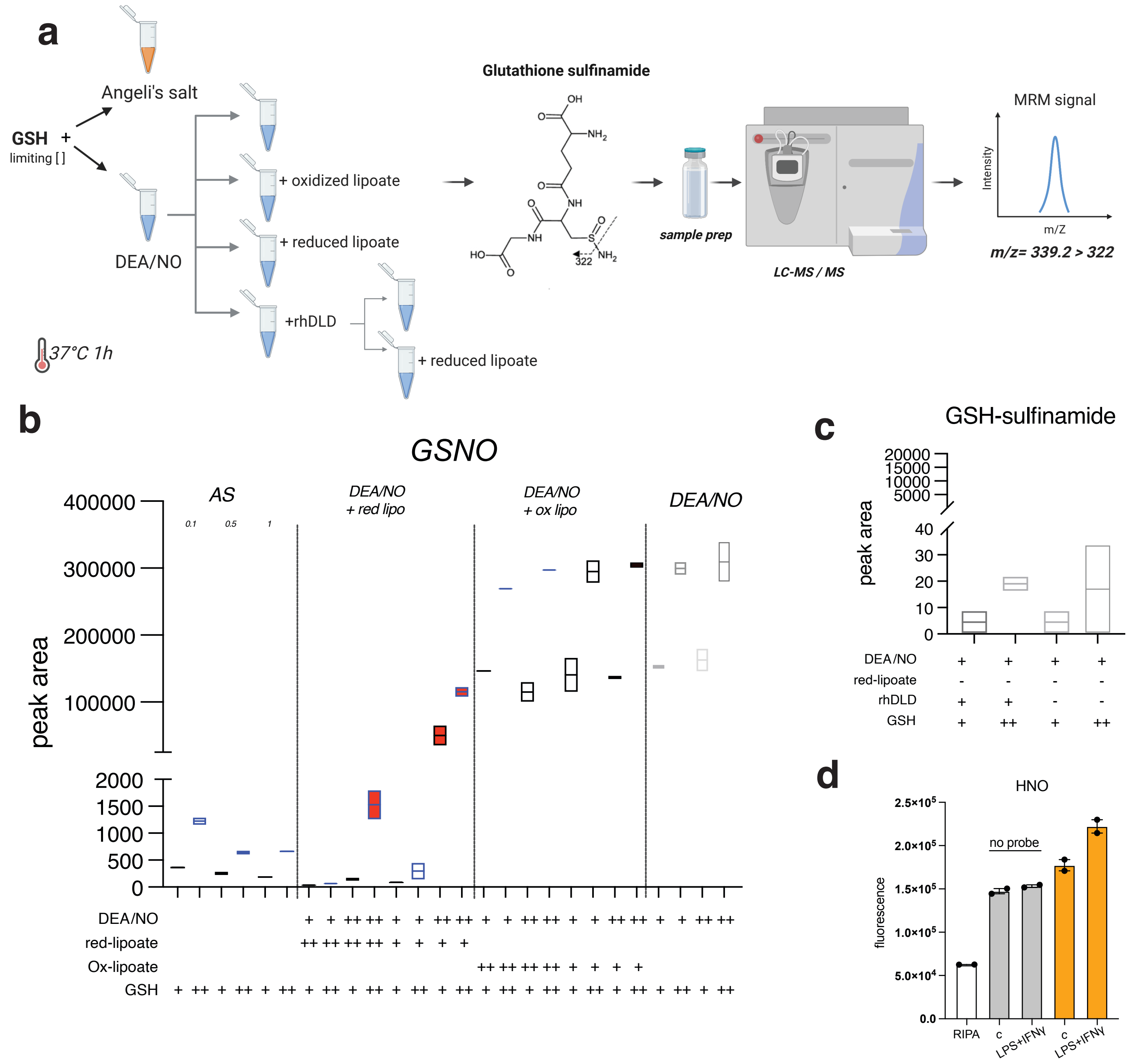

Supplementary Figure 3: a Experimental design for test tube reactions for detection of GSH-sulfinamide at LC-ESI/MS-MS. **b** Test tube reaction were carried out in the presence of GSH and AS or mixtures of either oxidized or reduced-lipoate with DEA/NO for 1h at 37°C. GSNO was quantified by ESI-LC/MS-MS. **c** Recombinant human DLD (rhDLD) was added to test tube reactions in absence of reduced lipoate and GSH-sulfinamide was quantified (n = 2 wells). Boxplots with floating bars (min to max) (**b** and **c)** are shown with line at mean. **d** WT BMDMs from male and female mice (mixed genders in all groups) were incubated with fluorescence probe for HNO or vehicle ctrl for 30 min and stimulated with LPS+ IFNγ for 4 h. After PBS washing, fluorescence was measured in total cell lysates (fluorescence or RIPA buffer alone is indicated) (n = 2 mice). All error bars display mean ± SEM. Data shown are representative of 2 or more independent experiments. Source data are provided as a Source Data file.

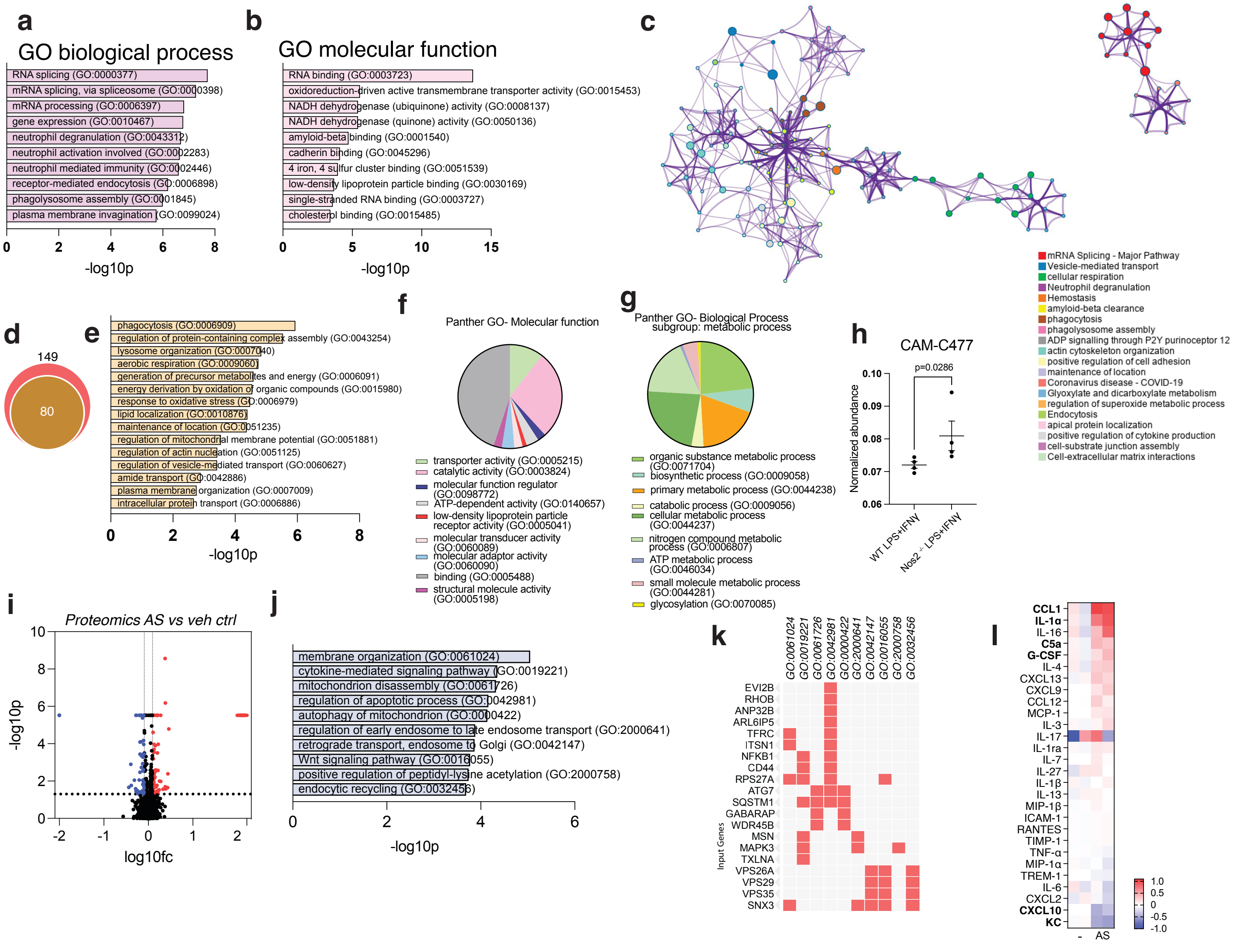

Supplementary Figure 4: a-b Pathway enrichment analysis (GO biological process and GO molecular function) on proteins from 4b (n = 4 mice). **c** Network plot of enriched terms, colored by cluster ID. **d** Venn diagram indicating number of stimulation-induced reduction-resistant proteins in WT BMDMs (from 4b) that are NOS2 dependent. **e** Total GO enrichment of proteins from S4d (protein number=80). **f-g** Enrichment in GO molecular function and "metabolic process" subgroup within GO biological process of proteins from S4d. **h** Normalized abundance of CAM-Cys^{477} containing peptide of DLD from broad TMT proteomics screen (4a) on mitochondrial lysates from WT and *Nos2*$^{-/-}$ BMDMs from male and female mice (mixed genders in all groups) stimulated with LPS+IFNγ (n = 4 mice). Data were analyzed with Mann-Whitney test (two-tailed). **i** Volcano plot of normalized abundance of total proteins from broad TMT proteomics screen (same as 4h) on mitochondrial lysates from *Nos2*$^{-/-}$ BMDMs stimulated with LPS+IFNγ and treated with AS (n = 4 mice). **j** Pathway enrichment analysis (GO biological process) on proteins from S4i with p<0.05, FC cutoff =1.25. **k** Clustergrammer visualization of enrichment analysis results from S4j displaying overlap of input gene list and gene lists of enriched terms. **l** Representative heatmap of the log2 fold change of secreted cytokines into the culture media when BMDMs from *Nos2*$^{-/-}$ male mice were stimulated as in 4h (n = 2 mice). Bold raws indicate significant changes (p<0.05) with cutoff FC=1.2. All error bars display mean ± SEM. Data shown are representative of 2 or more independent experiments. Source Data are provided as a Source Data file.

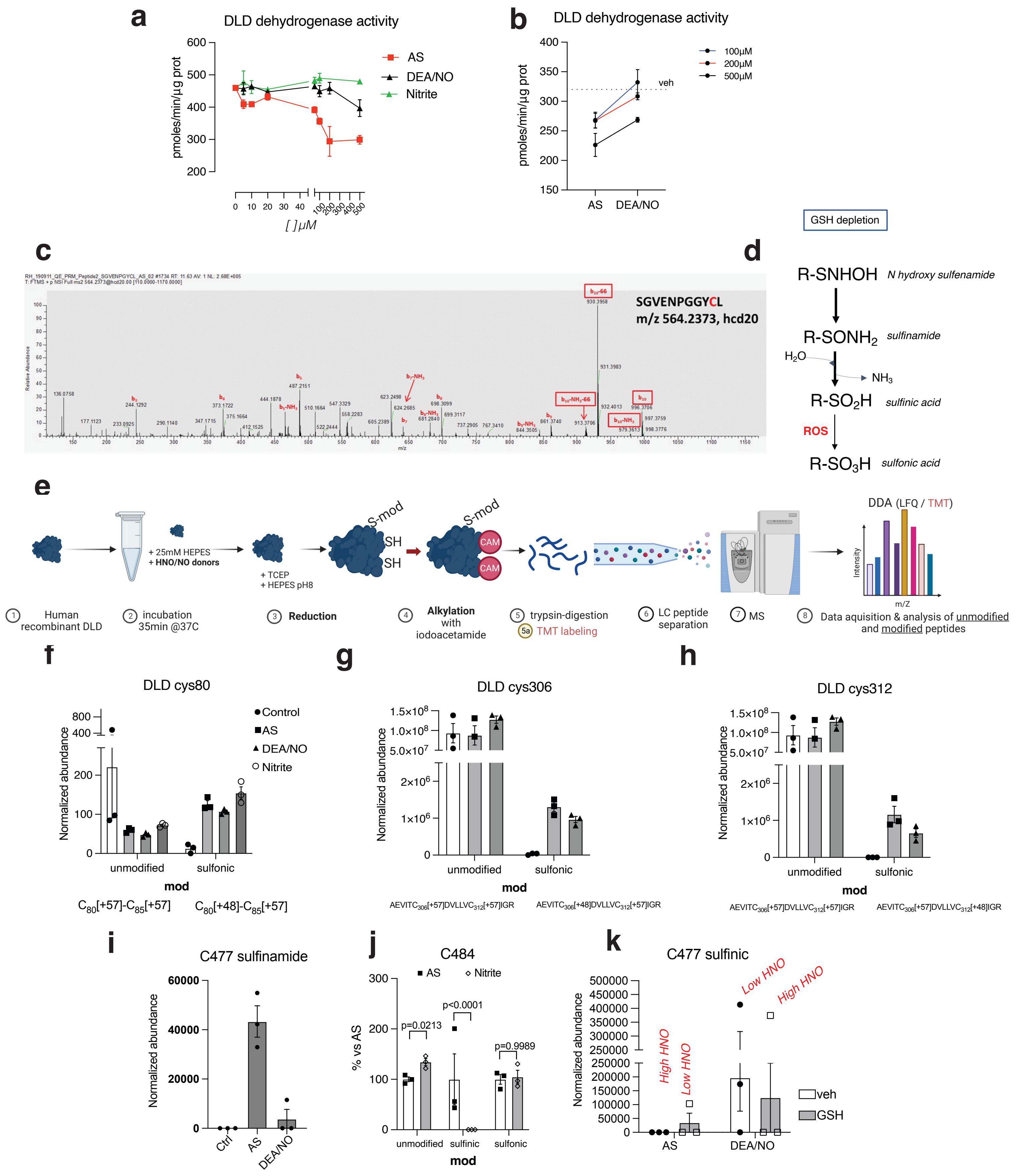

Supplementary Figure 5: a-b Dehydrogenase activity of rhDLD in the presence of AS, DEA/NO and $NaNO_2$ (Nitrite) at increasing concentrations (n = 5 wells). **c** Fragmentation pattern of Cys-containing synthetic peptide treated with AS consistent with presence of sulfinic acid modification. **d** Schematic illustration of mechanism of generation of sulfinamide and further oxidation statuses in condition of GSH depletion or high ROS. **e** Experimental design for proteomics studies on rhDLD incubated in vitro

with AS or DEA/NO. LFQ and TMT proteomics analysis of modifications of rhDLD at Cys^{80} (**f**), Cys^{306} (**g**) and Cys^{312} (**h**) in the presence of AS, DEA/NO or Nitrite (n = 3 wells). **i** TMT proteomics detection of Cys sulfinamide modifications of rhDLD at Cys^{477} in the presence of AS or DEA/NO (n = 3 wells). **j** TMT proteomics analysis (showed percentage relative to AS) of modifications of rhDLD at Cys^{484} in the presence of AS and Nitrite (n = 3 wells). Data were analyzed by two-way ANOVA (Sidak's post-tests). **k** LFQ proteomic quantification of Cys-sulfinic acid modifications of rhDLD at Cys^{477} in the presence of AS or DEA/NO and equimolar GSH (n = 3 wells). All error bars display mean ± SEM. Data shown are representative of 2 or more independent experiments. p values > 0.05 are reported as "ns" unless otherwise specified. Source data are provided as a Source Data file.

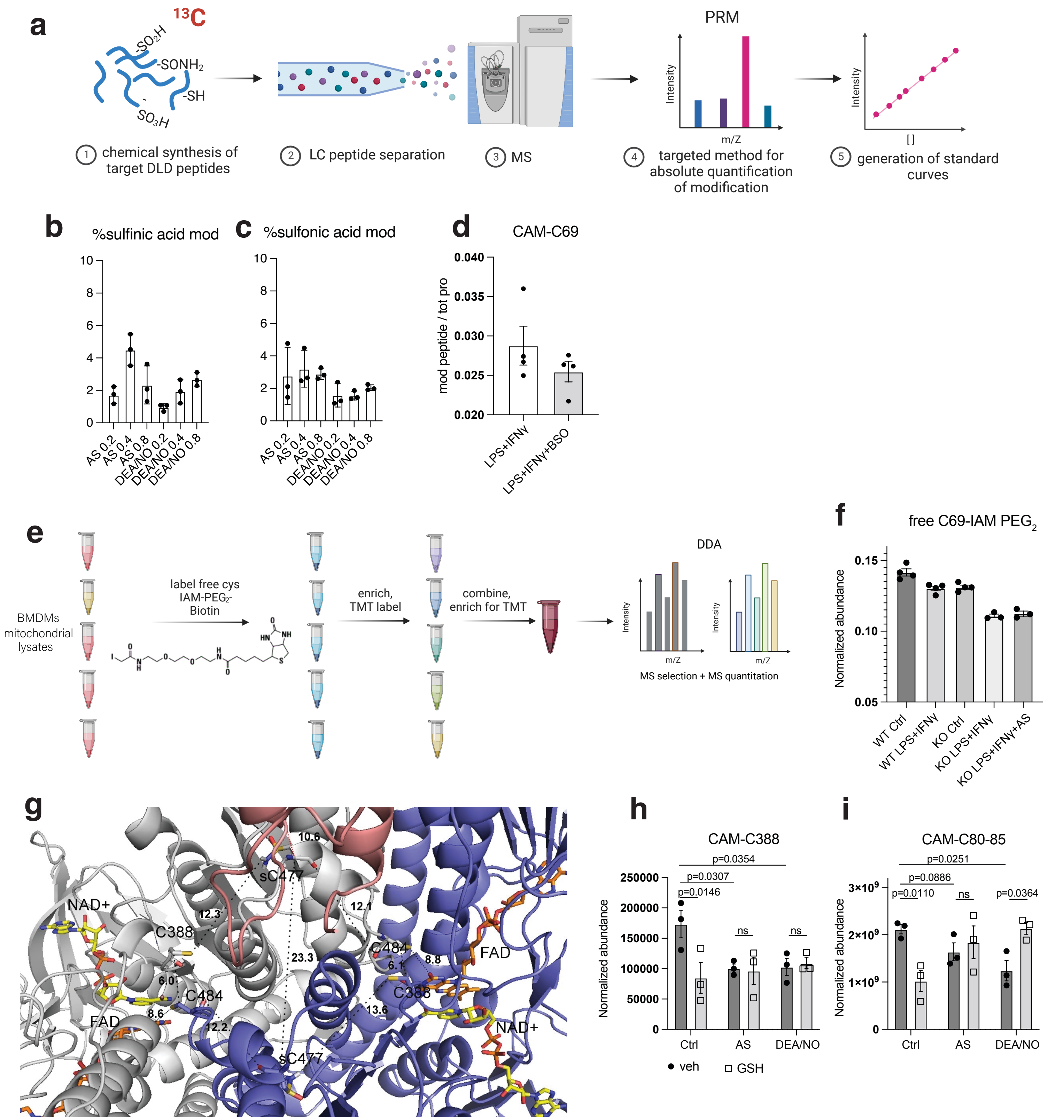

Supplementary Figure 6: **a** Experimental design for optimization of targeted proteomics studies on Cys[484] -containing peptides of DLD utilizing chemically synthesized and ^{13}C labeled housekeeping and bearing-modifications peptides; **b-c** Absolute quantification (% indicated) of portions of rhDLD modified at Cys[484] after incubation with AS or DEA/NO at increasing concentrations (n > 2 wells). **d** TMT proteomics assay on mitochondrial lysates from WT cells stimulated with LPS+ IFNγ and treated with BSO for 4h after ON stimulation, indicating normalized abundance of CAM- Cys[69] containing peptide (n = 4 mice). **e** Experimental design for proteomics on mitochodnrial lysates with a focus on free cysteines detection. **f** Quantification of IAM-PEG_2-enriched (free) Cys [69]-containing peptide of DLD in conditions from 5h-j (n > 3 mice). **g** Top view of dihydrolipoamide dehydrogenase (DLD_E3 dimer) in complex with the subunit binding of human dihydrolipoamide transacylase (E2b). DLD_E3

dimer is reported in grey (chain A) and blue (chain B) cartoon representation, whereas E2b is reported in pink cartoon representation. FAD and NAD+ are reported in orange and yellow sticks representation, respectively. Cysteine residues 484 and 388 are reported in sticks representation. Cys^{477} sulfinamide (sC477) in both chains is reported in sticks representation. The distance between the reported cysteine residues is labelled and indicated by black dashed lines. The distance between Cys^{484} and FAD is also indicated by black dashed lines and labelled. The distance between sC477 (aC) in chain A and K141 (aC) in DLD-E2b subunit is indicated by a red dashed line and labelled. **h-i** LFQ and TMT proteomic quantification of $Cys\text{-}^{388}$ CAM at $Cys^{80\text{-}85}$-CAM containing peptides of rhDLD in the presence of AS or DEA/NO and equimolar GSH (n = 3 wells). Data were analyzed by two-way ANOVA (Sidak's post-tests). All error bars display mean ± SEM. Data shown are representative of 2 or more independent experiments. p values > 0.05 are reported as "ns" unless otherwise specified. Source data are provided as a Source Data file.

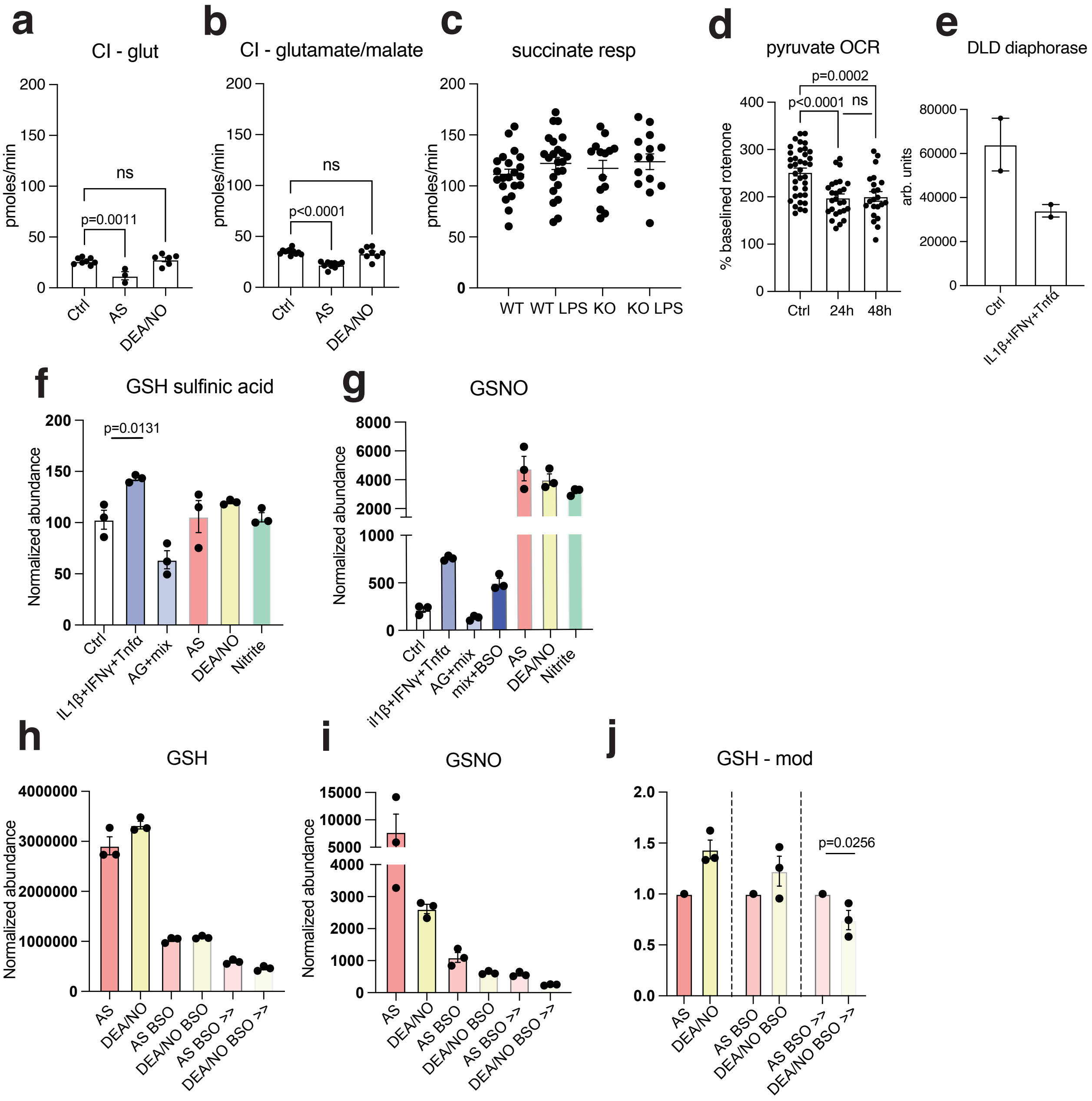

Supplementary Figure 7: a-b Studies of mitochondrial function on isolated liver mitochondria from male mice using electron flow assays performed as described in Methods. Bar graphs show quantified Complex I (OGDH-derived) respiration when glutamate alone or glutamate+malate were used as fuels (n > 8 wells). Data were analyzed by one-way ANOVA with Dunnet's multiple comparisons test. **c** Scatter plots show quantified succinate respiration as in 7d in mitochondrial livers from WT and *Nos2*$^{-/-}$ male and female mice (mixed genders in all groups) injected with LPS (n > 14 wells). **d** Bar graphs show quantified pyruvate respiration as in 7n, in primary hepatocytes stimulated with combination of cytokines at different time points (n > 21 wells). Data were analyzed by one-way ANOVA with Dunnet's multiple comparisons test. **e** Quantification of DLD in-gel diaphorase activity from 7o on mitochondrial extracts from primary hepatocytes isolated from male mice (n = 2 mice). GSH-sulfinic acid (**f**) and GSNO (**g**) were quantified by ESI-LC/MS-MS from primary hepatocytes cultures from male mice (n = 3 mice). **h-i** Primary hepatocytes from male mice where treated with AS or DEA/NO for total of 8h in the presence and absence of BSO for 4h at either 0.5 and 1mM: total cellular GSH and GSNO were quantified by ESI-LC/MS-MS (n = 3 mice). **j** Comparison of total modified GSH in conditions as in S7h-i in AS vs DEA/NO (% vs AS is shown) (n = 3 mice). Data were analyzed by unpaired t test (two-tailed). All error bars display mean ± SEM. Data shown and representative of 2 or more independent experiments. p values > 0.05 are reported as "ns" unless otherwise specified. Source data are provided as a Source Data file.

**Interaction energies (FoldX AnalyseComplex)**	**C477 sulfinamide modified DLD**	
Evaluated parameters	InterChain.A_B	InterChain.AB_E
Group1	A	AB
Group2	B	E
IntraclashesGroup1	29,5098	68,4145
IntraclashesGroup2	35,01	2,75887
**Interaction Energy**	**-90,6496**	**-9,56701**
Backbone Hbond	-20,4257	-1,89012
Sidechain Hbond	-40,3367	-6,84229
Van der Waals	-73,7061	-7,67289
Electrostatics	-8,97109	-2,11459
Solvation Polar	93,908	14,3893
Solvation Hydrophobic	-100,265	-9,20662
Van der Waals clashes	2,42316	0,206173
entropy sidechain	42,492	4,53423
entropy mainchain	15,6268	1,0743
torsional clash	1,47145	0,221082
backbone clash	14,7392	1,99328
helix dipole	-1,81962	-1,55306
electrostatic kon	-1,76226	-0,712563
energy Ionisation	0,715411	7,77E-16
Entropy Complex	2,384	2,384
Number of Residues	994	994
Interface Residues	195	29

Supplementary Table 1 Energy calculations about the investigated 3D models. “PDB.Chain” indicates the chain of the PDB used within the indicated analyses on the cited models obtained as described in the Methods section. Chain A and B indicate DLD_E3 chain within C477 sulfinamide modified protein. Chain E indicates the E2b subunit within the C477 sulfinamide modified protein. Bold numbers indicate interdomain interaction energies of energetically relaxed protein complexes.

**INTERFACEANALYZER APP**	**C477.sulfinamide.A_B**	**C477.sulfinamide.AB_E**
complex_normalized	0,961	1,021
**dG_separated**	**-160,118**	**-14,098**
dG_separated/dSASAx100	-2,014	-1,231
dSASA_hphobic	4426,353	603,668
dSASA_int	7946,799	1144,808
dSASA_polar	3520,446	541,139
delta_unsatHbonds	33	4
dslf_fa13	1,139	1,139
fa_atr	-6078,736	-6078,736
fa_dun	2756,953	2756,953
fa_elec	-1580,162	-1580,162
fa_intra_rep	12,371	12,371
fa_intra_sol_xover4	217,395	217,395
fa_rep	1906,902	1906,902
fa_sol	3821,457	3821,457
hbond_E_fraction	0,25	0,199
hbond_bb_sc	-150,074	-150,074
hbond_lr_bb	-238,149	-238,149
hbond_sc	-77,651	-77,651
hbond_sr_bb	-287,354	-287,354
hbonds_int	44	4
lk_ball_wtd	-57,06	-57,06
nres_all	940	991
nres_int	274	51
omega	167,863	167,863
p_aa_pp	-167,543	-167,453
packstat	0,603	0,603
per_residue_energy_int	0,526	0,903
pro_close	723,77	723,77
rama_prepro	170,234	170,234
ref	453,216	453,216
sc_value	0,668	0,504
side1_normalized	0,393	-0,176
side1_score	53,836	-5,117
side2_normalized	0,658	2,327
side2_score	90,154	51,187

Supplementary Table 2 The PDB.Chains used in the analyses with Rosetta were the same indicated in Table 1. A list of the energy terms taken from https://www.rosettacommons.org/docs/latest/application_documentation/analysis/interface-analyzer follows. Energy term abbreviation meanings: dslf_fa13 indicates disulfide geometry potential; fa_atr indicates Lennard-Jones attractive between atoms in different residues; fa_dun indicates the internal energy of sidechain rotamers; fa_elec indicates coulombic electrostatic potential with a distance-dependent dielectric; fa_intra_rep indicates Lennard-Jones repulsive between atoms in the same residue; fa_rep indicates Lennard-Jones repulsive between atoms in different residues; fa_sol indicates Lazaridis-Karplus solvation energy; hbond_bb_sc indicates sidechain-backbone hydrogen bond energy; hbond_lr_bb indicates backbone–backbone hbonds distant in primary sequence; hbond_sc indicates sidechain–sidechain hydrogen bond energy; hbond_sr_bb indicates backbone–backbone hbonds close in primary sequence; pro_close indicates Proline ring closure energy and energy of psi angle of preceding residue; rama indicates Ramachandran preferences; ref indicates reference energy for each amino acid; complex_normalized indicates the average energy of a residue in the entire complex; dG_separated, reported in bold characters, indicates the change in Rosetta energy when the interface forming chains are separated (binding energy), versus when they are complexed. dSASA_int, indicates the solvent accessible area buried at the interface, in square Angstroms. dG_separated/dSASAx100, separated binding energy per unit interface area × 100 to make units fit in score file. Scaling by dSASA controls for large interfaces having more energy; delta_unsatHbonds indicates the number of buried, unsatisfied hydrogen bonds at the interface; hbond_E_fraction indicates the amount of interface energy (dG_separated) accounted for by cross interface H-

bonds; hbonds_int indicates the total cross-interface hydrogen bonds found; nres_all indicates the total number of residues in the entire complex; nres_int indicates the number of residues at the interface; per_residue_energy_int; indicates the average energy of each residue at the interface; side1_score indicates the energy of one side of the interface; side2_score indicates the energy of the other side of the interface; side1_normalized indicates the average per-residue energy on one side of the interface; side2_normalized indicates the average per-residue energy on the other side of the interface. Rosetta energy terms are expressed in Rosetta Energy Units (REU) according to https://www.rosettacommons.org/docs/latest/rosetta_basics/Units-in-Rosetta .

Interaction energies (FoldX AnalyseComplex)	C484A		C484W	
Evaluated parameters	InterChain.A_B	InterChain AB_E	InterChain.A_B	InterChain.AB_E
Group1	A	AB	A	AB
Group2	B	E	B	E
IntraclashesGroup1	27,5645	66,2375	29,4462	73,2759
IntraclashesGroup2	34,9079	2,79891	34,9894	2,70199
**Interaction Energy (kcal/mol)**	**-90,4235**	**-8,39735**	**-88,4132**	**-8,8447**
Backbone Hbond	-20,6072	-1,89184	-20,5917	-1,89137
Sidechain Hbond	-38,3266	-6,76683	-38,7864	-6,8143
Van der Waals	-71,9259	-7,66656	-76,9798	-7,74808
Electrostatics	-8,34878	-2,10431	-9,24015	-2,15865
Solvation Polar	92,3531	14,4988	97,9562	14,663
Solvation Hydrophobic	-98,2408	-9,2003	-105,132	-9,26192
Van der Waals clashes	2,37661	0,196665	7,27147	0,208928
entropy sidechain	38,2173	5,44081	41,3872	5,42813
entropy mainchain	15,3262	1,04363	16,9557	0,701375
torsional clash	1,3884	0,214517	1,56881	0,223106
backbone clash	14,6115	1,98373	15,129	1,98614
helix dipole	-1,72891	-1,4439	-1,73476	-1,46441
electrostatic kon	-1,62149	-0,718011	-1,78866	-0,730549
energy Ionisation	0,71466	3,33067e-16	0,701271	3,33067e-16
Entropy Complex	2,384	2,384	2,384	2,384
Number of Residues	994	994	994	994
Interface Residues	192	29	196	29

Supplementary Table 3 Energy calculations about the investigated 3D models. Chain A and B indicate DLD_E3 chain within investigated mutants C484A or C484W). Chain E indicates the E2b subunit within the cited C484A and C484W mutants. Bold numbers indicate interdomain interaction energies of energetically relaxed protein complexes.
