# Supplementary material for: Pyruvate dehydrogenase operates as an intramolecular nitroxyl generator during macrophage metabolic reprogramming
Source: Nat Commun. 2023 Aug 22;14:5114. doi: 10.1038/s41467-023-40738-4 (PMC10444860; doi:10.1038/s41467-023-40738-4)
Supplement: Supplementary file 3 — Reporting Summary [file 41467_2023_40738_MOESM3_ESM.pdf]

nature portfolio

Corresponding author(s): Daniel McVicar

Last updated by author(s): Jul 11, 2023

Reporting Summary

Nature Portfolio wishes to improve the reproducibility of the work that we publish. This form provides structure for consistency and transparency in reporting. For further information on Nature Portfolio policies, see our Editorial Policies and the Editorial Policy Checklist.

Statistics

For all statistical analyses, confirm that the following items are present in the figure legend, table legend, main text, or Methods section.

n/a	Confirmed	
☐	☒	The exact sample size (*n*) for each experimental group/condition, given as a discrete number and unit of measurement
☐	☒	A statement on whether measurements were taken from distinct samples or whether the same sample was measured repeatedly
☐	☒	The statistical test(s) used AND whether they are one- or two-sided<br>*Only common tests should be described solely by name; describe more complex techniques in the Methods section.*
☐	☒	A description of all covariates tested
☐	☒	A description of any assumptions or corrections, such as tests of normality and adjustment for multiple comparisons
☐	☒	A full description of the statistical parameters including central tendency (e.g. means) or other basic estimates (e.g. regression coefficient) AND variation (e.g. standard deviation) or associated estimates of uncertainty (e.g. confidence intervals)
☐	☒	For null hypothesis testing, the test statistic (e.g. *F*, *t*, *r*) with confidence intervals, effect sizes, degrees of freedom and *P* value noted<br>*Give P values as exact values whenever suitable.*
☒	☐	For Bayesian analysis, information on the choice of priors and Markov chain Monte Carlo settings
☒	☐	For hierarchical and complex designs, identification of the appropriate level for tests and full reporting of outcomes
☒	☐	Estimates of effect sizes (e.g. Cohen's *d*, Pearson's *r*), indicating how they were calculated

Our web collection on statistics for biologists contains articles on many of the points above.

Software and code

Policy information about availability of computer code

Data collection	XF-96 analyzer (Seahorse Bioscience) was used to perform energetics studies.<br>Agilent 6410B (Agilent Technologies) interfaced with a 1200 Series HPLC quaternary pump (Agilent Technologies) with MassHunter Quantitative B.07.01.sp was used to acquire and quantify targeted mass spectrometry metabolite data.<br>Dionex U3000 RSLC in front of a Orbitrap Eclipse (Thermo) or Easy-nLC 1200 system in front of a Q-Exactive HF or Orbitrap Fusion (Thermo) were used to acquire proteomics data.<br>A 7890A GC system (Agilent Technologies) combined with a 5975C Inert MS system (Agilent Technologies) were used to measure global metabolite levels.<br>Agilent 6546 qTOF (Agilent Technologies) interfaced with a Agilent 1290 Infinity II ultra-high performance liquid chromatography (UHPLC) (Agilent Technologies) with MassHunter Profinder 8.0 was used to acquire and quantify targeted mass spectrometry metabolite data.
Data analysis	GraphPad Prism 9 was used to perform statistical analysis and plot results. Extracellular acidification rate (ECAR) and oxygen consumption rate (OCR) were analyzed by Wave (Agilent Technologies, Inc.). Proteomics data processing was carried out in Skyline 21.1, using either the sum of MS1 isotopic peak areas or the sum of fragment ion areas for each peptide PRM. Proteomics data were searched with Proteome Discoverer 2.4 using the Sequest node.<br>PyMOL 2.5 was used to build structural models. FOLDX4 and Rosetta Software were used for computational modeling and analysis of protein structures.<br>MassHunter Profinder 8.0 was used to process and quantitate 13C incorporation of target metabolites and evaluate isotopologue distribution.

For manuscripts utilizing custom algorithms or software that are central to the research but not yet described in published literature, software must be made available to editors and reviewers. We strongly encourage code deposition in a community repository (e.g. GitHub). See the Nature Portfolio guidelines for submitting code & software for further information.

March 2021

Data

Policy information about availability of data

All manuscripts must include a data availability statement. This statement should provide the following information, where applicable:

- Accession codes, unique identifiers, or web links for publicly available datasets
- A description of any restrictions on data availability
- For clinical datasets or third party data, please ensure that the statement adheres to our policy

All data supporting the findings of this study are available with the article, and can also be obtained from the corresponding author. Publicly available crystallized structure of the human DLD (PDB_ID: 3rnm) and hDLD crystallized in complex with FAD and NAD+ (1zmd.pdb) were used in this study. The mass spectrometry proteomics data have been deposited to the ProteomeXchange Consortium via the MASSive partner repository under accession code MSV000092336. Source data are provided with this paper.

Human research participants

Policy information about studies involving human research participants and Sex and Gender in Research.

Reporting on sex and gender	N/A
Population characteristics	N/A
Recruitment	N/A
Ethics oversight	N/A

Note that full information on the approval of the study protocol must also be provided in the manuscript.

Field-specific reporting

Please select the one below that is the best fit for your research. If you are not sure, read the appropriate sections before making your selection.

[x] Life sciences [ ] Behavioural & social sciences [ ] Ecological, evolutionary & environmental sciences

For a reference copy of the document with all sections, see nature.com/documents/nr-reporting-summary-flat.pdf

Life sciences study design

All studies must disclose on these points even when the disclosure is negative.

Sample size	3-9 mice per group were used for animal studies. No prior sample size calculation was performed. Group sizes for animal studied were established considering previous experience with yields of cell numbers and biological matrices. No statistical methods were used to predetermine sample size. Sample sizes were determined based on prior studies with similar experimental design and on the known variability of the assay, balancing statistic robustness with resource availability. The number of mice that were used for each experiment was determined to give reliable and robust conclusions. The number of mice per group for each experiment are indicated in figure legends, and depicted as individual dots in graphs. Animal experiments were repeated at least three times.
Data exclusions	No data exclusions were made.
Replication	Results of experiments are based on representative results of at least 3 independent bone marrow derived macrophage cultures (for in vitro experiments)/cohorts of mice (for in vivo experiments) following the same protocol. All findings were replicated successfully.
Randomization	Animals were randomized by age for bone marrow extractions. For in vivo studies, mice were also randomized by bodyweight and sex prior to challenge with LPS. For in vitro experiments samples were randomly allocated into experimental groups.
Blinding	Investigators were blinded as to the experimental groups mice were allocated. Only animal technical staff who were performing the injections were aware of the allocation of mice to each group. Investigators performing analysis of samples for mass spectrometry (proteomics and metabolomics) were blinded to the allocation of mice. Investigators were not blinded for the in vitro experiments because the treatment groups were labeled, however all experiments were objective and conclusions based on multiple technical replicates and statistical significance.

Reporting for specific materials, systems and methods

We require information from authors about some types of materials, experimental systems and methods used in many studies. Here, indicate whether each material, system or method listed is relevant to your study. If you are not sure if a list item applies to your research, read the appropriate section before selecting a response.

Materials & experimental systems

n/a	Involved in the study
☐	☒ Antibodies
☐	☒ Eukaryotic cell lines
☒	☐ Palaeontology and archaeology
☐	☒ Animals and other organisms
☒	☐ Clinical data
☒	☐ Dual use research of concern

Methods

n/a	Involved in the study
☒	☐ ChIP-seq
☒	☐ Flow cytometry
☒	☐ MRI-based neuroimaging

Antibodies

Antibodies used

anti-Mouse antibodies for immuno blot:
from R&D systems: Human/Mouse/Rat PDHX Affinity Purified Polyclonal Ab (#AF6014, RRID:AB_1964669).
from Millipore: Rabbit Anti-Lipoic Acid Polyclonal Antibody, Unconjugated (#437695, RRID:AB_212120)
from Santa Cruz Biotechnology: Mouse monoclonal anti-TOM20 (#sc-136211, RRID: AB_2207538)
from Origene: Mouse monoclonal anti DDK (#TA50011-100, RRID:AB_2622345)
from abcam: Mouse monoclonal anti Actin (#ab6276, RRID:AB_2223210)
also used for immunoprecipitation: Anti-Pyruvate Dehydrogenase E2 (Abcam) (#ab172617, RRID:AB_2827534), Recombinant Anti-Lipoamide Dehydrogenase (Abcam)(#ab133551, RRID:AB_2732908)

Validation

Validation information can be found on the manufacturers' websites using the antibody Cat#:
https://www.abcam.com/primary-antibodies/how-we-validate-our-antibodies
https://www.emdmillipore.com/US/en/life-science-research/antibodies-assays/antibodies-overview/Antibody-Development-and-Validation/cFOb.qB.8McAAAFOb64qQvSS,nav
https://www.origene.com/products/antibodies/quality
https://www.rndsystems.com/products/human-mouse-rat-pdhx-antibody_af6014
https://www.scbt.com/it/p/tom20-antibody-29

Eukaryotic cell lines

Policy information about cell lines and Sex and Gender in Research

Cell line source(s)

Human embryonic Kidney cells (HEK293T) were purchased from ATCC

Authentication

no authentication was performed

Mycoplasma contamination

line was tested negative for mycoplasma contamination

Commonly misidentified lines
(See ICLAC register)

none

Animals and other research organisms

Policy information about studies involving animals; ARRIVE guidelines recommended for reporting animal research, and Sex and Gender in Research

Laboratory animals

C57BL/6J and NOS2 deficient mice (B6.129P2-Nos2tm1Lau/J purchased from the Jackson Laboratory) were maintained and bred in the Frederick National Laboratory Core Breeding Facility, aged 8-10 weeks at start of experiments. Dark/light cycle is 12/12 (6a-6p). Temperature 68-79F and humidity 30-70%.

Wild animals

Wild animals were not used

Reporting on sex

Sex based analyses were not performed.

Field-collected samples

Field collected animals were not used

Ethics oversight

Mice were used in accordance with an approved protocol by the NCI Frederick Institutional Animal Care and Use Committee

Note that full information on the approval of the study protocol must also be provided in the manuscript.
